# In the Neighborhood: Metabolic Outcomes among Residents Exposed to Aircraft Noise

**DOI:** 10.1289/ehp.122-A193

**Published:** 2014-07-01

**Authors:** Carol Potera

**Affiliations:** Carol Potera, based in Montana, has written for *EHP* since 1996. She also writes for *Microbe*, *Genetic Engineering News*, and the *American Journal of Nursing*.

Exposure to transportation noise from roadways and airports is an environmental stressor that has been associated with disrupted sleep, release of stress hormones such as cortisol, elevated blood pressure, increased risk for cardiovascular disease, and disruption of glucose metabolism.^1^^,^^2^ In this issue of *EHP*, researchers at the Karolinska Institute explore the relationship between long-term exposure to aircraft noise and metabolic outcomes including body mass index (BMI), waist circumference, and type 2 diabetes.^3^

Past studies of transportation noise include the Hypertension and Exposure to Noise near Airports (HYENA) study, which reported a higher prevalence of hypertension with increasing exposure to night-time aircraft noise among residents near six European airports.^4^ In another study of 6 million Medicare enrollees living near 89 U.S. airports, increased noise exposure was associated with more hospital admissions for cardiovascular disease.^5^ Exposure to road traffic noise near homes also was associated with increased risk for diabetes in a Danish study of 57,053 people aged 50–64, who were followed for about 10 years.^6^

**Figure:**
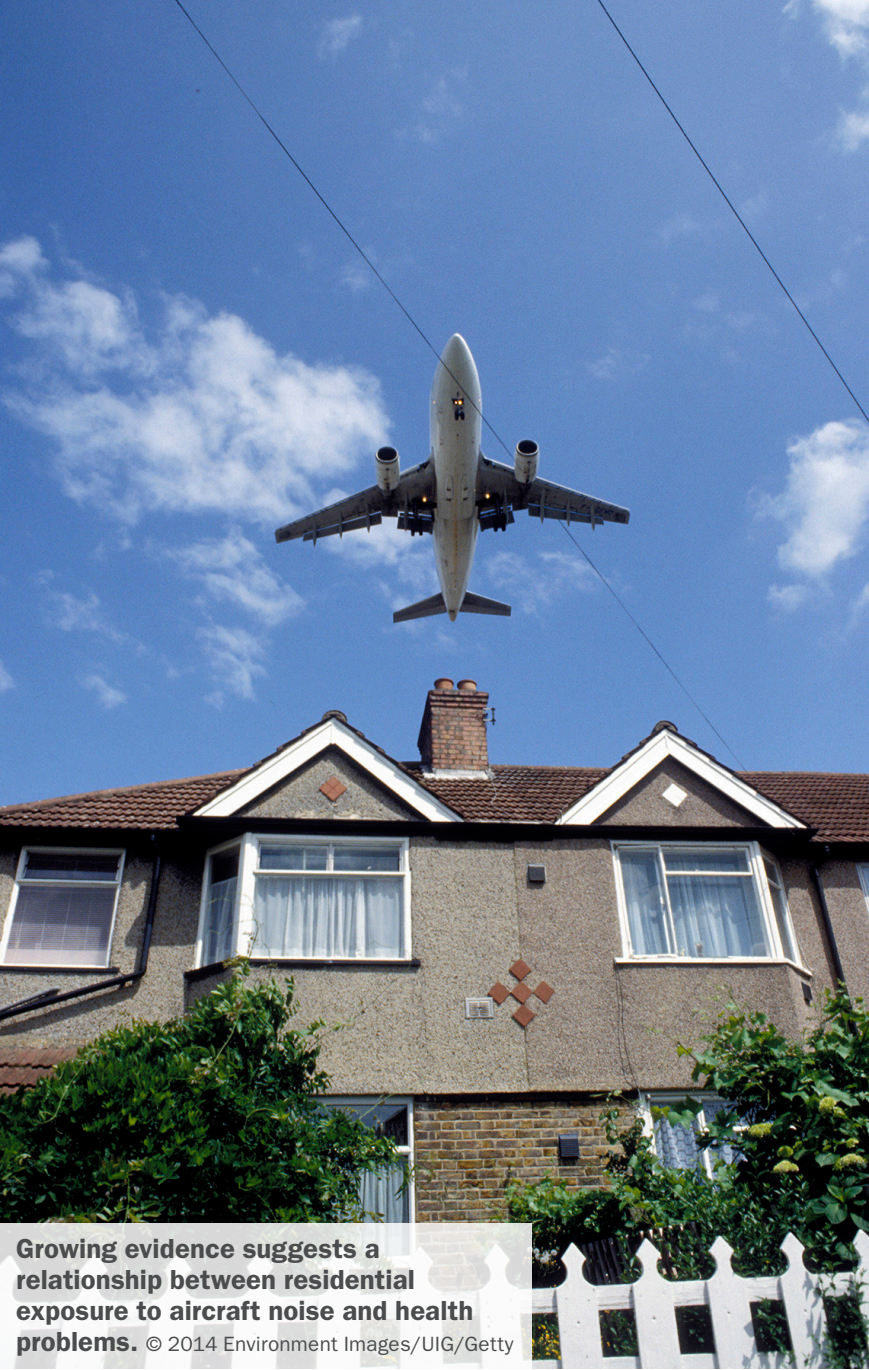
Growing evidence suggests a relationship between residential exposure to aircraft noise and health problems. © 2014 Environment Images/UIG/Getty

In the current study, the researchers monitored 3,128 men and 4,821 women living in Sweden’s Stockholm County. Participants were 35–56 years old at the study’s start, and they were followed for 8–10 years. Half had a family history of diabetes, but all had normal oral glucose tolerance tests when the study began. About 9 years later, followup testing identified 412 cases of prediabetes and 159 cases of type 2 diabetes. Exposure to aircraft noise from nearby Stockholm Arlanda Airport was estimated based on participants’ residential addresses and modeled noise data from the Swedish Airports and Air Navigation Services.

The investigators report that each 5-db(A) *L*_den_ increase in aircraft noise was associated with an average 1.51-cm increase in waist circumference, most prominently among people who did not move during the study period. The association remained after adjustments for socioeconomic and lifestyle factors, but BMI and the development of type 2 diabetes were not consistently associated with aircraft noise. Previously, this team reported an association between exposure to aircraft noise and increased risk for hypertension in these participants.^2^

“We cannot make any clear conclusions regarding diabetes since there were not enough cases,” says study leader Charlotta Eriksson. An observed association between aircraft noise and diabetes among women was based on just 12 individuals with high noise exposure.

However, the results fit with the hypothesis that aircraft noise stimulates the release of cortisol. This stress hormone contributes to central obesity, which is measured by waist circumference, whereas BMI is a marker for general obesity, notes Eriksson. But because this is the first known study to indicate a potential link between aircraft noise and markers of central obesity, Eriksson says the results should be interpreted with caution.

It remains to be determined whether road traffic noise, which is a far more common exposure than aircraft noise, shows a similar relationship with waist circumference, BMI, or diabetes. If such relationships were to be determined, “the consequences of long-term exposure to traffic noise may be of even greater importance than previously anticipated,” Eriksson says. “From a public health viewpoint, it is increasingly important to consider noise in the urban planning process to minimize the number of people exposed.”

The study by Eriksson and colleagues “adds to the growing literature examining associations between noise and cardiovascular outcomes, moving beyond hypertension to additional … pathways,” says Jonathan Levy, associate chair of the Department of Environmental Health at Boston University. He says the lack of a strong connection to BMI and diabetes “points to the need for larger cohort studies to investigate noise-related cardiovascular events and multiple putative mechanisms.”
